# Microencapsulated Diets as an Alternative to Bivalve Feeding: Particle Size and Microalga Content Affect Feed Intake

**DOI:** 10.3390/ani13122009

**Published:** 2023-06-16

**Authors:** Vitória Pereira, Sílvia F. S. Pires, Andreia C. M. Rodrigues, Pearl Ofoegbu, Pedro Bem-Haja, Amadeu M. V. M. Soares, Luís E. C. Conceição, Rui J. M. Rocha, Mário Pacheco

**Affiliations:** 1 CESAM—Centre for Environmental and Marine Studies and Department of Biology, University of Aveiro, 3810-193 Aveiro, Portugal; 2 CINTESIS@RISE—Center for Health Technology and Services Research, Department of Education and Psychology, University of Aveiro, 3810-193 Aveiro, Portugal; 3 Sparos, Lda., 8700-221 Olhão, Portugal; 4 Riasearch, Lda., 3870-168 Murtosa, Portugal

**Keywords:** bivalve mollusks, microalgae, food intake, feeding selectivity

## Abstract

**Simple Summary:**

Bivalve shellfish aquaculture represents a sustainable and nutritionally sound path to provide food for a growing global population. New advances in feed formulation, namely microencapsulation, demonstrated great potential to face key bivalve nutrition problems, enabling increased quality production as the lack of naturally occurring food may result in non-lethal prejudice with losses in organoleptic characteristics. To test the applicability of algae-based microencapsulation, this study evaluated the food intake in five bivalve species that are highly valuable and widely cultivated throughout the world: Pacific oyster, grooved razor shell, carpet shell clam, manila clam, and common cockle. Four microencapsulated diets were implemented, incorporating two different microalgae species into two different diameter pellets. Overall, all the tested diets were easily ingested by the bivalves, although species-related profiles of food intake depending on the pellet size were observed. These results enabled a better selection of feed with appropriate profiles, offering a margin of opportunity to improve bivalve nutritional value and contributing to reinforcing knowledge in the framework of shellfish aquaculture. Moreover, a combination of different microalgae could be explored in the future as it may provide good sources of essential nutrients and a better product for the final consumer.

**Abstract:**

Bivalve mollusks represent a nutritious source with a low environmental impact; as a result, they are one of the most attractive aquaculture options. Advances in microencapsulation technology offer great potential to face key bivalve nutrition problems, and an alga-based microencapsulated diet can turn enriched bivalves into potential functional foods. The central goal of this study was the evaluation of food intake as a function of particle size and microalga content following the supply of four microencapsulated diets, incorporating as core material *Nannochloropsis* sp. or *Tetraselmis* sp. in 20 or 40 µm diameter pellets (diets N20, T20, N40, and T40, respectively) in five bivalve species (*Magallana gigas*, *Solen marginatus*, *Ruditapes decussatus*, *Ruditapes philippinarum*, and *Cerastoderma edule*). Overall, all tested diets were easily ingested, although food intake was higher for N20 (except for the *S. marginatus*, which showed a higher rate for the diet T40). Concerning a size-related analysis, *C. edule* and *S. marginatus* favored, respectively, smaller and bigger pellet-sized diets, with no signs of selectivity for microalga species. The diet T20 was the lesser ingested, except for *C. edule*. This knowledge enables a better selection of feed with appropriate and species-adjusted profiles, contributing to the optimization of microencapsulated diets for bivalve rearing and a better final product.

## 1. Introduction

Shellfish farming is the most profitable sector of European marine aquaculture [1]. Moreover, bivalve mollusks offer one of the most attractive options for meeting sustainability goals in the context of global food production, since they represent a nutritious and low-impact source with minimal addition to the environment, increasing demand for both fresh and processed products [1,2,3].

Bivalves are opportunistic feeders that exploit the diverse nature of suspended particulate matter and have a feeding process of active sorting, where particles of different sizes and shapes are ingested by filtration [4,5]. Food particles are mostly collected through the inhalant siphon and sorted on the labial palps and gills, and unwanted particles (particularly sharp-edged particles) are rejected in pseudo-feces [6], except for oysters, a non-siphoned species. Oysters are well adapted through their pumping behavior and particle selection activity [7], using the gills to stick the particles and then move them up to the mouth [8].

Studies on natural food availability for bivalves in coastal waters reveal marked short-term to seasonal variations in concentration, composition, and nutritional value [9]. As nutrition can be the dominant factor influencing bivalve growth [10], prolonged starving periods by lack of naturally occurring food may result in non-lethal prejudice to nutritional composition, with losses in organoleptic characteristics of the final product [11,12,13,14]. Based on their biochemical profile and suitable size for ease of digestion, live microalgae are an important natural food source for bivalves [15], supplying the necessary nutrition for the whole growth cycle [16,17]. Microalgae are also used in experimental systems and their importance is recognized in the depuration process [18], where bivalves are kept under conditions that maximize the natural filtering activity to allow the expulsion of intestinal contents and subsequent removal of microbial contaminants [19].

*Nannochloropsis* sp. and *Tetraselmis* sp. are microalgae that can be produced on a large scale at a relatively low cost and are rich in eicosapentaenoic acid (EPA), an important long-chain omega-3 (ω-3), and polyunsaturated fatty acids (PUFAs), promoting an increase in shell length and the total weight of bivalves [20,21,22]. However, the problem of microalgae mass production for feeding bivalves is still very challenging. The efficient culturing and growth to improve the economic viability of bivalve production need further development [16], and multiple flaws in live microalgae feed increase costs and may compromise animal growth. To ensure reasonably stable culture quality, microalgae must be grown in high-intensity artificial lighting with temperature and air control systems, creating an expensive and major bottleneck to the bivalve-rearing segment [2,23].

In an attempt to provide cost-effective alternative feeding strategies, substitution products, such as bacteria, yeast, dehydrated algae, slurry, paste of algae, and various microcapsule formulations have been tested previously [24,25]. A microencapsulated feed consists of a shelf-stable formulation of nutritional and functional components surrounded by a digestible capsule that can be optimized for maximum ingestion and cost advantage, efficiently delivering high-quality nutrients, disease control agents, or quality enhancers to bivalves [2,6]. Microcapsules, as artificial diets, must fulfill a series of criteria such as maximal availability in the water column, minimal loss of nutrients during administration, good palatability, acceptable digestibility, and balanced nutrient composition [26].

Despite the restrictions of microencapsulation technology in scaling up due to the high cost, new advances offer great potential to face key nutrition problems by overcoming the limitations associated with the selection of the appropriate strain and adjusted particle size when different microalgae were mixed. Microparticle size can be tailored to bivalve species or life stages, and buoyancy can be optimized to ensure that they remain within reach [2,26], minimizing nutrient leaching to water, while also being sterile and not a disease vector [23]. This approach has a great advantage over nutrient delivery systems such as freeze-dried algal powders, which tend to float and can clump into particles too large to be accessed by bivalves [2]. Mass production of microcapsules is simple and thus circumvents conventional feed wastage costs [23]. However, there is little information regarding microcapsules incorporating microalgae, the relative efficiency of microparticle retention by bivalves, and its preferential particle size.

Using five bivalve target species (within commercial size) that are highly valuable and widely cultivated throughout the world, namely, Pacific oyster (*Magallana gigas*), grooved razor shell (*Solen marginatus*), carpet shell clam (*Ruditapes decussatus*), manila clam (*Ruditapes philippinarum*), and common cockle (*Cerastoderma edule*), the central goal of this study was the evaluation of food intake as a function of particle size and microalga content (core material) following the supply of four microencapsulated diets, incorporating *Nannochloropsis* sp. or *Tetraselmis* sp. in 20 or 40 µm diameter pellets for 24 h. This approach ultimately intended to reinforce knowledge of new ecotechnological solutions in the framework of bivalve aquaculture.

## 2. Materials and Methods

### 2.1. Bivalve Species and Experimental Design

Adult wild specimens of Pacific oyster (*Magallana gigas*), grooved razor shell (*Solen marginatus*), carpet shell clam (*Ruditapes decussatus*), manila clam (*Ruditapes philippinarum*), and common cockle (*Cerastoderma edule*) with legal commercial size and average body weights of 63.14 ± 6.82, 24.30 ± 6.60, 7.48 ± 1.57, 17.00 ± 3.81, and 8.14 ± 2.95 g, respectively, were collected (in July) at Ria de Aveiro, Portugal, a bivalve growing area.

**Figure 1:**
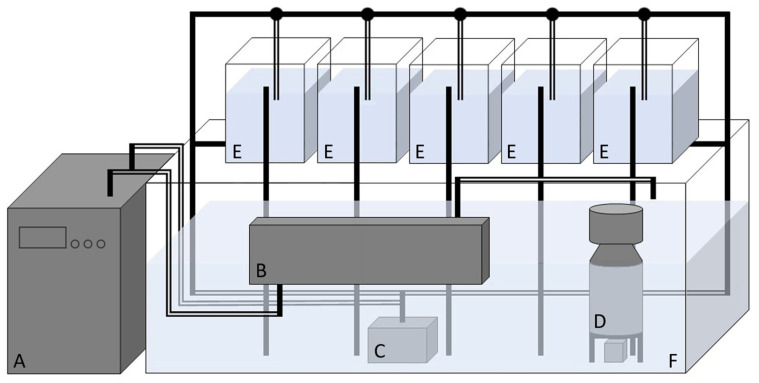
Schematic representation of the recirculation system and experimental tanks replicated for each experimental diet: A—chiller, B—UV-c disinfection system, C—recirculation pump, D—protein skimmer, E—10-L tanks (one for each species), and F—250-L tank. The full dashes correspond to PVC tubes, the double dashes correspond to hoses, and the circles to taps.

Following harvesting, live bivalves were transported (3 h) in net bags at ambient air temperature directly to the laboratory in Aveiro, Portugal, and placed into the recirculation systems using artificial seawater (ASW). ASW was prepared by dissolving an appropriate mix of salts (Red Sea Salt—Red Sea) in potable filtered tap water (filtering system—Equation, BCM-Bricolage, Carnaxide, Portugal). Each species batch was placed in a monolayer 10-L capacity tank connected to a 250-L capacity tank, acting as a recirculating system (filled with 150 L of ASW). The recirculation system was replicated 4 times (pseudoreplication), 1 for each experimental diet, and was equipped with a UV-c system (Vecton V2 600, 25 W, 6.000 µW·s/cm^2^, TMC Iberia, São Julião do Tojal, Portugal), chiller (HAILEA, model HC-300A, Chaozhou, China), recirculating submerged water pump (Eheim, Typ 1262 210, 3400 l/h), and protein skimmer (EHEIM, Typ 1103 800, Deizisau, Germany) (see Figure 1). Water temperature was maintained at 15 ± 1 °C and salinity at 35 ± 1 during the whole trial and was checked using a multi-parameter measuring instrument (WTW, KS MultiLine^®^ 2, 2F0004, Weilheim, Germany). To allow acclimation, increase the appetite, and avoid disturbing their endogenous feeding rhythm, a fasting period of 6 days was carried out prior to the feeding trial.

The water circuit starts with its impulsion through the pump (C) from the large tank (F) to the chiller (A) then to the UV-c system (B) and returns to the same tank. The water is also continuously pumped through the circulation system to the 10-L tanks (E), with the flow being controlled by the taps. In parallel with this process, the protein skimmer (D) acts independently, removing proteins and other organic particles dissolved in the water. During the 24-h feeding experiment, the UV-c system and the protein skimmer were turned off.

For each experimental treatment, at time zero, 3 g of the specific microencapsulated diet (see Section 2.2) was provided per 150 L of ASW, corresponding to 0.02 g/L. Then, 3 sampling times were considered for food intake estimation (see Section 2.3) at 2, 5, and 24 h after the introduction of the diets in the different systems (times one, two, and three, respectively). At each point in time, 3 oysters (n = 3) and 5 individuals from the other 4 species (n = 5) were sampled per experimental group, and soft tissues were excised, weighted, immediately frozen in liquid nitrogen, and stored at −80 °C until analyses.

### 2.2. Experimental Diets

Four different microencapsulated diets, produced by spray-drying technology by SPAROS, Lda. (Olhão, Portugal), were prepared by incorporating *Nannochloropsis* sp. or *Tetraselmis* sp. microalgae together with a marine oil rich in phospholipids in 20 or 40 µm diameter particles, using gelatin as an excipient. The gelatin was firstly dissolved in water at 50 °C. The remaining ingredients were subsequently added, and the water volume was adjusted to obtain a solids percentage of 15%. The suspension was maintained at 50 °C and then atomized using a GEA Niro Spray Dryer, Model Mobile Minor (GEA, Switzerland) under the following conditions: inlet temperature 160 °C, outlet temperature 85–90 °C, air pressure 6 bar, flow rate 10 mL/min. The resulting particles had almost neutral buoyancy, sinking slowly after breaking the surface tension. From now on, the diets are represented by the abbreviations N20, N40, T20, and T40, where the letters N and T represent, respectively, *Nannochloropsis* and *Tetraselmis*, and the numbers express the pellet size in µm. The spherical microcapsules also incorporate a fluorescent marker (Solvent Red 196) for later quantification of the food intake. The mixtures to be microencapsulated where thoroughly mixed in a high-shear homogenizer to ensure a homogeneous mixture, including the suspension of the very small particles of the insoluble fluorescent marker in water. *Nannochloropsis* sp. and *Tetraselmis* sp. microalgae were provided by NECTON S.A. (Olhão, Portugal), a microalgae production company. The respective diet formulations are presented in Table 1.

### 2.3. Food Intake Estimation through Fluorescence Analysis

#### 2.3.1. Tissue Preparation

The whole soft tissues of each animal were individually homogenized by sonication (Branson Sonifier 250, Emerson Electric Co., St. Louis, MI, USA) on ice using 6 mL of ultra-pure water and were placed in tubes at −80 °C until analyses.

#### 2.3.2. Rapid Screening Fluorescence

The rapid screening fluorescence assay was performed based on the method applied by Masias et al. [27] in black 96-well microplates (Greiner Bio-One, Frickenhausen, Germany). For the fluorescence determination, each well contained a final volume of 300 μL of blanks, standards, or homogenized tissues. The fluorescence was measured using a fluorescence plate reader (F-7000 Fluorescence Spectrophotometer, Hitachi, Japan) with an excitation wavelength of 475 nm and an emission wavelength of 645 nm. Each determination was performed in quadruplicate using a standard curve of the microencapsulates in water. Food intake was calculated for each animal as mg diet consumed/g animal (fresh weight) through the formula:

- Diet concentration in mg diet/mL solution

$$\frac{\left(sample\;fluorescence-blank\right)-b}{m}$$

where *b* and *m* correspond to values from the standard curve.

- Volume adjustment and final diet concentration in mg diet/g animal

$$\frac{\frac{\left(diet\;concentration\;*\;Vi\right)}{Vf}*d}{sample\;weight}$$

where *Vi* corresponds to the initial sample volume (homogenate), *Vf* to the well volume, and *d* to the dilution of the sample.

Though mean values for individual food intake were also depicted, the representation of the final results privileged the estimated marginal means of food intake considering the experimental model design [3 (time) × 4 (diet)] and interactions between them. In fact, observed means do not account for the effect of other factors in the model.

### 2.4. Statistical Analyses

Analyses were run in R 2.8 software [28] and data sets were verified for normality through the Shapiro–Wilks test and homogeneity of variance through Levene’s test to fulfill statistical demands. Considering non-normal distribution inferential analysis, data were log transformed before being submitted to inferential statistical analysis. The pattern of results was the same for the transformed and untransformed scores; therefore, descriptive statistics are based on the untransformed data for easy interpretation. Results were expressed as estimated marginal means for violin plots and as mean ± standard error (SE) for individual food intake values.

Considering the hierarchical structure of the data (multilevel), the variability of samples per condition, and the absence of equal time intervals between times, a linear mixed model was performed for each species and fitted by REML and the Satterthwaite method for degrees of freedom. The model explored for all species can be expressed using the following formula:

$$Y_{ti}=\beta_{0}+\beta_{1}\left(TIME\right)+\beta_{2}\left(DIET\right)+\beta_{3}(TIME)(DIET)+b_{0i}+\varepsilon_{ti}$$

where $Y_{ti}$ is the food intake at time *t* for individual *i*, $\beta_{0}$ is the mean intercept, $\beta_{1}$ is the mean growth rate, ${TIME}_{ti}$ is the value of predictor for individual *i* at time *t* (e.g., expected change in food intake as function of 1 time change in time variable), $b_{0i}$ is the random effect and allows the intercepts (food intake at time 1, i.e., at 2 h) to vary across individuals, i.e., the individual heterogeneity, and $\varepsilon_{ti}$ is a time-specific residual. A *p* < 0.05 significance level was considered for all cases [29].

## 3. Results

### 3.1. Pacific Oyster (Magallana gigas)

**Figure 2:**
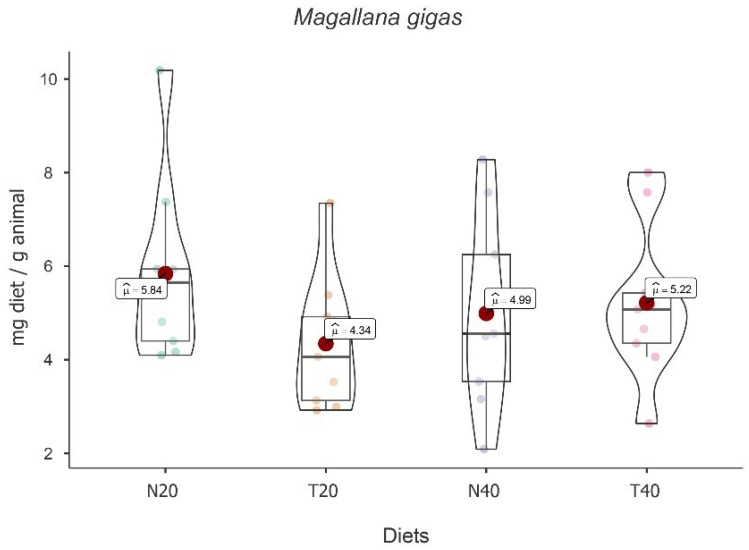
Violin plot (box plots with probability density of the data at different values) with estimated marginal means of food intake (red dot) in pacific oysters (*Magallana gigas*) for the diets N20, T20, N40, and T40. Mean values are also provided.

No main effect of the diet [*F*(3, 24) = 2.049, *p* = 0.134] was observed. Despite the absence of statistical differences, descriptively, the highest mean food intake value was found for diet N20, followed by diets T40, N40, and T20 (see Figure 2).

On the other hand, the results showed the main effect of time [*F*(2, 24) = 17.037, *p* < 0.001], with a decrease in food intake over time. In fact, paired-wise *t* tests followed by Bonferroni correction showed significant differences between times [t_2-1_(16) = −2.222, *p* = 0.002 and t_3-1_(16) = −2.964, *p* < 0.001]. Simple main effects showed that there is a significant variation over time for diets N20 [*F*(2, 24) = 4.84, *p* = 0.017], N40 [*F*(2, 24) = 6.81, *p* = 0.005], and T40 [*F*(2, 24) = 3.73, *p* = 0.039] (see Table 2).

No interaction effect between time and diet was recorded [*F*(6, 24) = 0.540, *p* = 0.772], showing that food intake over time does not depend on the diet.

### 3.2. Grooved Razor Shell (Solen marginatus)

**Figure 3:**
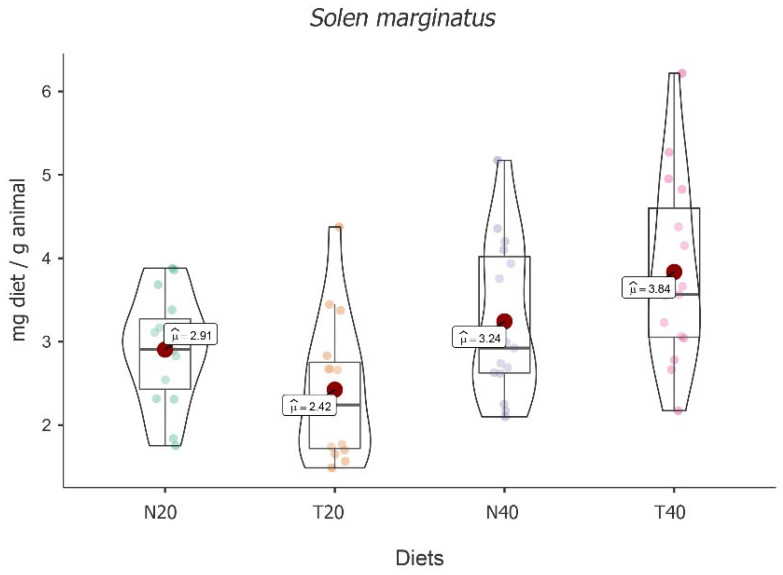
Violin plot (box plots with probability density of the data at different values) with estimated marginal means of food intake (red dot) in grooved razor shells (*Solen marginatus*) for the diets N20, T20, N40, and T40. Mean values were also provided.

A significant fluctuation was recorded between the four diets in this study [*F*(3, 16) = 5.829, *p* < 0.001]. The highest mean food intake value was obtained for diet T40 (M = 3.84; 95% CI [3.31, 4.36]), followed by diets N40 (M = 3.24; 95% CI [2.72, 3.76]) and N20 (M = 2.91; 95% CI [2.38, 3.43]). The lowest value was found for diet T20 (M = 2.42; 95% CI [1.90, 2.94]) (see Figure 3).

Despite these absolute differences, post hoc tests followed by Bonferroni correction showed that significant differences were only registered between the higher value of the diet T40 compared to diet T20 [t(16) = −4.061, *p* < 0.01].

Regarding time, no main effect was observed [*F*(2, 32) = 2.09, *p* = 0.140], with no variability between times. Despite the effect of diet, an interaction between time and diet was recorded [*F*(6, 32) = 2.71, *p* = 0.03] (see Table 3).

Simple main effects of food intake showed significant fluctuations among diets for time one (2 h) [*F*(3, 46) = 4.34, *p* < 0.01] and for time three (24 h) [*F*(3, 46) = 6.64, *p* < 0.001]. However, multiple comparisons between diets followed by Bonferroni correction showed no statistically significant differences between pairs of diets for time one. Regarding time three, the comparisons between T20 vs. N40 [t(46) = −3.93, *p* = 0.019 ] and T20 vs. T40 [t(46) = −3.66, *p* = 0.04] at 24 h showed significant differences, with T20 being the lowest value in both cases. On the other hand, simple effects showed that there is no significant variation over time for any diet. Nevertheless, diets T20 and T40 varied the most, with results that, although not significant (*p* value = 0.087 and 0.088, respectively), configured a trend.

### 3.3. Carpet Shell Clam (Ruditapes decussatus)

**Figure 4:**
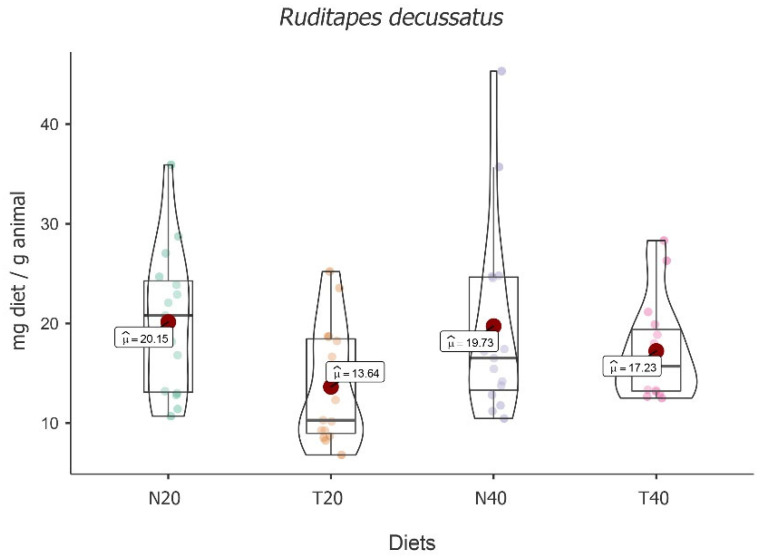
Violin plot (box plots with probability density of the data at different values) with estimated marginal means of food intake (red dot) in carpet shell clams (*Ruditapes decussatus*) for the diets N20, T20, N40, and T40. Mean values were also provided.

Significant fluctuation between the four diets under study was observed [*F*(3, 16) = 7.73, *p* = 0.002]. This species demonstrated a higher mean food intake for diet N20 (M = 20.15; 95% CI [2.80, 3.08]), followed by N40 (M = 19.73; 95% CI [2.75, 3.03]), T40 (M = 17.23; 95% CI [2.67, 2.95]), and T20 (M = 13.64; 95% CI [2.39, 2.67]) (see Figure 4).

Despite the absolute differences, post hoc tests followed by Bonferroni correction showed that significant differences were only registered between diet T20 and all other diets, i.e., T20 vs. N20 [t(16) = 4.412, *p* = 0.003], T20 vs. N40, [t(16) = −3.848, *p* = 0.009] and T20 vs. T40 [t(16) = −3.044, *p* < 0.05].

The main effect of time was also observed [*F*(2, 32) = 24.46, *p* < 0.001], with an increase in food intake over time. In fact, comparisons followed by Bonferroni correction showed significant differences between times [t_3-2_(32) = 4.90, *p* < 0.001 and t_3-1_(32) = 6.77, *p* < 0.001]. The interaction effect between time and diet [*F*(6, 32) = 4.48, *p* = 0.002] demonstrates that food intake over time depends on the diet (see Table 4).

Simple effects showed significant variation over time for all diets, except for T40 [*F*(2, 32) = 5.92, *p* = 0.007, *F*(2, 32) = 14.59, *p* < 0.001 and *F*(2, 32) = 15.56, *p* < 0.001, for N20, T20 and N40, respectively]. Differences were also identified between diets for all times [*F*(3, 48) = 2.92, *p* = 0.043, *F*(3, 48) = 9.19, *p* < 0.001 and *F*(3, 48) = 4.60, *p* = 0.007, for times one, two, and three, respectively]. After multiple comparisons followed by Bonferroni correction, the comparisons between diets N20 and T20 [t(48) = 4.56, *p* = 0.002] and T20 and T40 [t(48) = −4.21, *p* = 0.007] for time two (5 h) stand out the most with higher values for N20 and T40, as well as between diets N40 and T40 [t(48) = 3.61, *p* = 0.048] for time three (24 h), with N40 showing the highest value.

### 3.4. Manila Clam (Ruditapes philippinarum)

**Figure 5:**
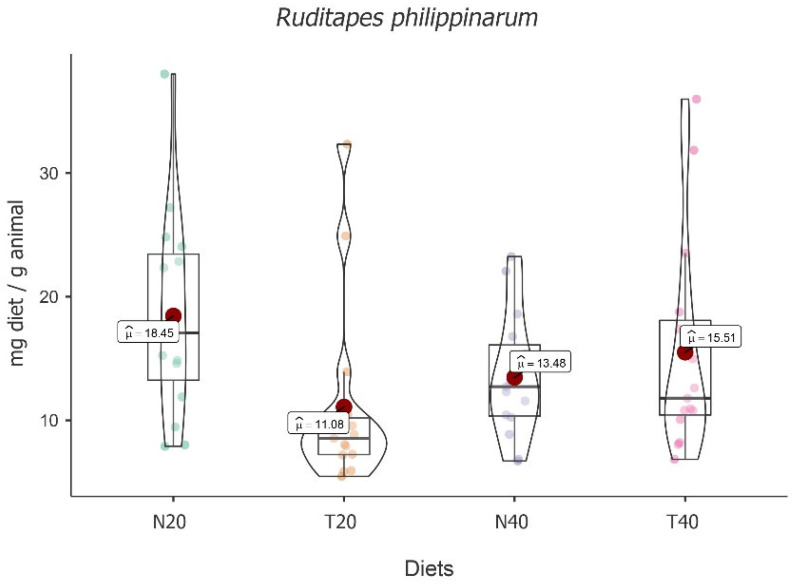
Violin plot (box plots with probability density of the data at different values) with estimated marginal means of food intake (red dot) in manila clams (*Ruditapes philippinarum*) for the diets N20, T20, N40, and T40. Mean values were also provided.

The highest mean food intake value was obtained for diet N20 (M = 18.45; 95% CI [2.66, 2.98]), followed by T40 (M = 15.51; 95% CI [2.46, 2.78]), N40 (M = 13.48; 95% CI [2.38, 2.70]), and T20 (M = 11.08; 95% CI [2.10, 2.42]), with significant fluctuation among diets [*F*(3, 48) = 8.26, *p* < 0.001] (see Figure 5).

Despite the absolute differences, post hoc tests followed by Bonferroni correction showed that significant differences were only registered between diets N20 and T20 [t(16) = 4.904, *p* < 0.001] and diets T20 and T40 [t(16) = −3.140, *p* = 0.04], with T20 showing the lowest value.

The results also showed an increase in food intake over time [*F*(2, 48) = 32.10, *p* < 0.001] with significant differences between times, i.e., t_2-1_(32) = 4.01, *p* = 0.001, and t_3-2_(32) = 4.00, *p* = 0.001. However, there was no interaction effect between time and diet [*F*(6, 48) = 1.70, *p* = 0.142], showing that, despite some variability, food intake over time does not depend significantly on the diet (see Table 5).

Although there was no interaction effect, considering the exploratory character of the present study and the conceptual interest, we performed simple diet effects moderated by time, recording a significant difference at time two (5 h) [*F*(3, 48) = 9.00, *p* < 0.001]. This result is explained by the difference between N20 and T20 [t(48) = 5.18, *p* < 0.001].

### 3.5. Common Cockle (Cerastoderma edule)

**Figure 6:**
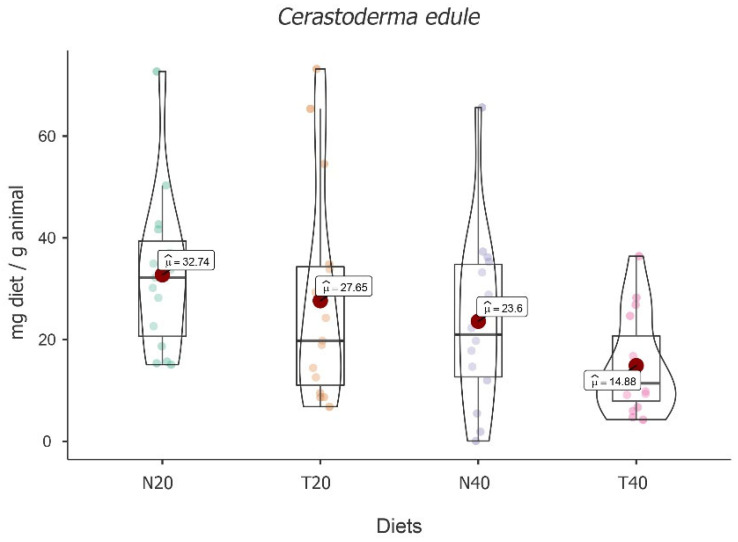
Violin plot (box plots with probability density of the data at different values) with estimated marginal means of food intake (red dot) in common cockles (*Cerastoderma edule*) for the diets N20, T20, N40, and T40. Mean values were also provided.

The results displayed the main effect of diet [*F*(3, 47) = 7.42, *p* < 0.001], showing a significant fluctuation among the diets under study. The highest mean food intake value was obtained for diet N20 (M = 32.74; 95% CI [4.97, 6.19]), followed by diet T20 (M = 27.65; 95% CI [4.31, 5.53]) and then diet N40 (M = 23.6; 95% CI [3.62, 4.89]). The lowest value was found for diet T40 (M = 14.88; 95% CI [3.06, 4.28]) (see Figure 6).

Despite these absolute differences, post hoc tests followed by Bonferroni correction showed that significant differences were only recorded between diets N20 and N40 [t(16) = 1326, *p* < 0.05] and between diets N20 and T40 [t(15) = 4.46, *p* = 0.003], with N20 showing the highest value.

The results also showed the main effect of time [*F*(2, 47) = 11.84, *p* < 0.001], with an increase in food intake over time and significant differences between times [t_3-2_(31) = 2.64, *p* = 0.039 and t_3-1_(32) = 4.85, *p* < 0.001]. An interaction effect between time and diet was also recorded [*F*(6, 47) = 7.03, *p* < 0.001], showing that food intake over time depends on the diet (see Table 6).

Simple effects showed that there is significant variation over time for all diets [*F*(2, 47) = 4.02, *p* = 0.025, *F*(2, 47) = 9.90, *p* < 0.001, *F*(2, 47) = 14.32, *p* < 0.001, and *F*(2, 47) = 3.71, *p* = 0.032, for N20, T20, N40, and T40, respectively]. On the other hand, differences between diets were only identified for time one [*F*(3, 47) = 5.54, *p* = 0.002] and for time three [*F*(3, 47) = 14.18, *p* < 0.001]. After multiple comparisons followed by Bonferroni correction, the comparisons between N20 and N40 [t(47) = 3.86, *p* = 0.023] stand out for time one, and also for time three between T40 and all other diets (all *p* values < 0.001).

## 4. Discussion

There are several microalgae species cataloged for bivalve feeding; however, despite the general recognition that a mixed microalgal diet gives improved nutritional values, its use is limited by the selection of algal strain, suitable particle size, and nutritive composition to obtain the whole expected benefit [17,21]. To overcome this problem, substantial research has been devoted to developing artificial diets, and some advances have been made, particularly with microencapsulated diets [6,23]. As microalgae are the natural food of filter-feeding bivalves, a preparation of microencapsulated algae would offer the advantage over maintenance expenditure of algal culture systems, and the practical advantage of being readily and reliably available [6]. There is also a great potential for microencapsulated feeds to offer an efficient way to deliver replacement or supplementary diets to bivalves that can tackle problems with feed and disease, enabling increased quality production output and industry growth [2,6]. Quality enhancers, such as flavorings, can also be incorporated, which could strengthen consumer demand for a diet with more sustainable seafood [2].

In the food industry, spray drying is the most used microencapsulation method due to the emulsifying and film-forming properties of gelatin [30]. Gelatin is a good carrier for many food ingredients and forms thermally reversible gels with water, giving gelatin products unique organoleptic properties, including flavor release [30,31]. However, to be ingested, microcapsules need to have characteristics meeting bivalves’ acceptability in terms of density, shape, buoyancy, content (as microalgae surface properties), and size. In the present study, only the influence of size and content was evaluated once the other factors were kept constant among the diets.

Most suspension-feeding bivalves can capture particles greater than 5 μm in diameter with nearly 100% efficiency [32,33,34]. Depending on the species, bivalve larvae tend to select particles smaller than 10 µm diameter, while juvenile and adult bivalves are likely to select particles ranging up to 20 µm [35]. The maximum size of ingested particles is not clearly established; yet, considering a spherical particle, several studies reported an efficient uptake of particles with diameters as high as 200–300 μm [34,36]. Concerning the food intake dependence on pellet size, the current results pointed out higher levels of ingestion of 20 µm compared with 40 µm particles by four of the five species addressed (*M. gigas*, *R. decussatus*, *R. philippinarum*, and *C. edule*), which can be related to the greater similarity with natural food size, such as microalgae (approx. 2–25 µm), as corroborated by Gui et al. [35]. *M. gigas*, a non-siphoned species, did not differ from the siphoned ones, as already demonstrated by Nielsen et al. [37], who concluded that algae in the size range of 7–32 µm were retained with 100% efficiency by the same species. However, only two of the bivalve species demonstrated independence between particle size and microalga species incorporated. Thus, *C. edule* favored the intake of smaller pellets (20 µm), contrary to *S. marginatus* which displayed higher levels of ingestion of larger pellets (40 µm). This result may be associated with the size of the specimens used, assuming that, among the studied siphoned species, *C. edule* had the smallest size/weight and *S. marginatus* the largest.

Suspension-feeding species, such as bivalves, have developed various strategies for controlling their food intake process. Differences in the characteristics of the habitat, particularly food availability and quality, give rise to adjustments, allowing species to maintain adequate levels of energy acquisition. These mechanisms allow them to manage the amount of ingested food and the production of pseudo-feces, which enhances the nutritive value of ingested particles and optimizes energy gain. This way, these bivalves can sort and ingest particles of interest [34,38]. Food particles are collected through the inhalant siphon and sorted on the labial palps and gills depending on physical attributes, such as size and shape, while unwanted particles, particularly those that are sharp-edged or inorganic, are preferentially rejected in pseudo-feces. Once ingested, food particles are swept along ciliated sorting areas in the stomach to the digestive glands, and any particles too large for digestion are then rejected down deep grooves in the stomach to the intestine [4,6]. In the oyster, a non-siphon filter feeder, this process occurs partly in the gills but mainly in the palps. Waterborne particles, upon striking the gills, are entangled in mucus and carried by the cilia of the gill epithelium. The mucus-covered particles are then carried to the posterior margins of the palps. At this point, the material may either pass between the grooved and ciliated faces of the palps and then toward the mouth or be pushed off onto the ventral mantle at the edges of the palps. Then, the mouth will push the particles to the stomach [8,39]. At comparable body weights, inhalant current velocities of the oyster *M. gigas* were lower than in the siphoned species, while modeled exhalant jets were higher but oriented horizontally instead of vertically as in other species [40]. Notwithstanding, all species in this study were separated in the feeding trial. As all adult species were of a commercial size, oysters did not have a comparable body weight to the other species.

The duration of the experiment was based on Houki et al. [41], which reported that an optimum feeding cycle depends on energy saving as well as food availability. Bivalves may close their shells soon after they obtain enough food; therefore, a 24-h experiment seems enough to meet the present goals and ensure minimal animal use. The number of sampling times was currently restricted to three, since shellfish feeding behavior can be easily disturbed by the effects of cascades, aeration, and, especially, by operator handling, ceasing to function effectively and impairing the experiment [42]. The intermediate two- and five-hour sampling times gave a more accurate measure and a guarantee that the animals had eaten since the time zero. It was ensured that the quantity of food in the experimental system was not a limiting factor throughout the trial for all species. Although a decrease in food intake over time was observed for *M. gigas*, the other four species demonstrated the opposite profile, with *S. marginatus* showing no main effect of time with no variability between times, and *R. decussatus*, *R. philippinarum* and *C. edule* displaying a main effect of time with an increase in food intake over time, regardless of the diet.

Prior studies have revealed that bivalves display selective feeding regarding nutrient content and are able to discriminate between different microalgal species to reject those of low quality [43]. This selection was mediated by interactions between lectins found in the mucus of feeding organs and microalgae physicochemical surface properties, as carbohydrates, the degree of wettability, and surface charge [43,44]. Although microcapsule wall materials prevented physical contact between alga cells and bivalve feeding organs, Espinosa et al. [34] reported that animals can still distinguish the microcapsule content and confirmed that the oyster *M. gigas* significantly ingested one diet and preferentially rejected the other by testing two different encapsulated diets. The same authors stated that extracellular metabolites produced by microalgae play a crucial role in the pre-ingestive selection of particles.

The current microalgae choice relies on the fact that *Nannochloropsis* species (order Eustigmatales) may occur in both freshwater and marine environments and are known to contain a high concentration of polyunsaturated fatty acids (PUFAs), such as EPA (38.39%) [22]. They are used in aquaculture to feed a wide variety of marine organisms [20]. They are unicellular and planktonic, with either a 2–4 μm diameter subspherical or 3–4 × 1.5 μm cylindrical cells [45]. A species of *Nannochloropsis* genus was also found to be rich in phytosterols, cholesterol [46], carotenoids, polyphenols, and vitamins [47], and can be produced on a large scale at a low cost [20]. *Nannochloropsis* sp. microencapsulates appear to be the most suitable diet for most of the studied bivalve species. This result is in agreement with Yamasaki et al. [20], who found out that a *Nannochloropsis* sp.-enhanced diet promotes a significantly greater average shell length of *R. philippinarum*, a shortened rearing time, and provides a stable supply of algal diet. *Nannochloropsis* species have also been used for several decades to produce nutraceuticals and feed supplements, for both humans and animals [48,49]. In fact, the richness of bioactive compounds in these microalgae has long inspired their exploitation for human nutrition, where daily consumption of a few grams would result in a regular dietary intake of essential molecules for the prevention of some pathologies or disorders [47]. On the other hand, *Tetraselmis* spp., order Chlorodendrales, are unicellular flagellates with elliptical or almost spherical, slightly flattened cells with an invagination at the anterior end from which arise four equal flagella in two opposite pairs. *Tetraselmis* species differ considerably in cell size and shape. Cells can be round, ovoid, elliptical, flattened, compressed, or a combination of these shapes, in which their side lengths vary from 3.5—25 µm [45,50]. Marine and freshwater *Tetraselmis* species are also known, and they are easy to culture and can be easily mass produced [51]. According to Abbas et al. [17], *Tetraselmis* sp. had high carbohydrate contents and high bivalve conditioning potential, being a good diet for *M. gigas* [34] and *R. decussatus* [52]. *Tetraselmis* sp. microencapsulates, highly ingested by *S. marginatus* in this study, is corroborated by Fernández-Tajes et al. [53], as they also used it to feed *S. marginatus* during their experimental trial. This microalga species also contains many active pigments such as carotenoids, with strong antioxidant and protective activities on human cells, as well as a potential cosmeceutical interest [54].

Microalgae are likewise used in several industries, with an emphasis on their application in biofuel production, human health, and beauty [46,55]. Recently, the search for phytosterols for human health application has gained increased attention. Some marine organisms, such as shellfish, are rich in phytosterols, particularly bivalves as microalgae consumers, making them marine products with healthy functional properties [46]. Therefore, feeding bivalves with microencapsulates with a combination of different microalgae could also be explored, as it may have different nutritional profiles that could provide good sources of essential nutrients, necessary for their growth and development. This can also be achieved through the feed introduction in a bivalve depuration context, as it can improve their nutritional status and provide a better product for the final consumer, unveiling a possible line of development for the present research.

Bearing in mind that this study was based on adult specimens of a commercial size, all from Ria de Aveiro, the possibility that animals from other origins may show somewhat different food intake patterns should not be undervalued, due to the determinant role of environmental factors associated to the history of the animals.

## 5. Conclusions

Overall, all the tested diets were easily ingested by the bivalve species addressed, either siphoned or non-siphoned, as indicated by intake estimation. Food intake data revealed that the diet with higher acceptability was that composed of *Nannochloropsis* sp. microencapsulates with a 20 µm diameter pellet size, except for *S. marginatus* (which showed higher intake for *Tetraselmis* sp. microencapsulates with 40 µm diameter pellets). *Tetraselmis* sp. microencapsulates with a 20 µm diameter pellet size promoted a lesser intake for all bivalve species, except for *C. edule*.

Concerning a size-related analysis, *C. edule* and *S. marginatus* favored, respectively, smaller and bigger pellet-sized diets, with no signs of selectivity for microalga species.

Though the study concerns a short feeding period (24 h) and complementary longer experiments would be convenient in the near future, this information is useful in the field of nutrition of aquacultured bivalves, enabling a more sustained choice of microencapsulated algal feeds with appropriate and species-adjusted profiles in terms of pellet size and core material.

## Figures and Tables

**Table 1:** Ingredients, proximate composition, and essential fatty acid content of the experimental diets.

	N20	T20	N40	T40
**Pellet size (** **µm)**	ø 20	ø 20	ø 40	ø 40
**Ingredients (%DM)**				
*Nannochloropsis* sp.	79	-	79	-
*Tetraselmis* sp.	-	79	-	79
Gelatin	15	15	15	15
MPL 40	5	5	5	5
Marker	1	1	1	1
**As fed basis (%DM)**				
Protein	46.20	33.81	46.20	33.81
Fat	17.70	9.93	17.70	9.93
Ash	10.82	25.89	10.82	25.89
DHA	1.06	1.06	1.06	1.06
EPA	4.29	0.44	4.29	0.44
C18:3n-3	0.05	0.05	0.05	0.05
C18:2n-6	0.07	0.07	0.07	0.07

Estimated composition values based on analyzed ingredient composition. DM—dry matter; MPL 40—marine phospholipids 40%.

**Table 2:** Mean values of food intake (mg diet/g animal) in pacific oysters (*Magallana gigas*) at the different sampling times (2, 5, and 24 h, corresponding to times one, two, and three, respectively). Values are expressed as mean ± SE.

		Time (h)		
		2	5	24
Diets	N20	7.74 ± 1.08	4.94 ± 0.43	4.84 ± 0.45
	T20	5.88 ± 0.61	3.82 ± 0.42	3.33 ± 0.30
	N40	6.80 ± 0.93	5.24 ± 0.43	2.93 ± 0.35
	T40	6.88 ± 0.75	4.41 ± 0.73	4.36 ± 0.14

**Table 3:** Mean values of food intake (mg diet/g animal) in grooved razor shells (*Solen marginatus*) at the different sampling times (2, 5, and 24 h, corresponding to times one, two, and three, respectively). Values are expressed as mean ± SE. Statistically significant differences (*p* < 0.05) between diets within the same time are expressed by letters to represent pairs of diets.

		Time (h)		
		2	5	24
Diets	N20	2.86 ± 0.17	2.38 ± 0.24	3.47 ± 0.19
	T20	2.84 ± 0.39	2.64 ± 0.33	1.79 ± 0.09 a,b
	N40	2.96 ± 0.29	2.90 ± 0.34	3.86 ± 0.42 a
	T40	4.44 ± 0.59	3.34 ± 0.32	3.72 ± 0.35 b

**Table 4:** Mean values of food intake (mg diet/g animal) in carpet shell clams (*Ruditapes decussatus*) at the different sampling times (2, 5, and 24 h, corresponding to times one, two, and three, respectively). Values are expressed as mean ± SE. Statistically significant differences (*p* < 0.05) between diets within the same time are expressed by letters to represent pairs of diets.

		Time (h)		
		2	5	24
Diets	N20	14.37 ± 1.77	20.68 ± 1.30 a	25.39 ± 3.59
	T20	9.77 ± 0.65	10.29 ± 1.51 a,b	20.87 ± 1.30
	N40	14.80 ± 0.96	13.37 ± 1.07	31.02 ± 3.72 c
	T40	14.31 ± 0.71	20.40 ± 2.86 b	16.99 ± 1.18 c

**Table 5:** Mean values of food intake (mg diet/g animal) in manila clams (*Ruditapes philippinarum*) at the different sampling times (2, 5, and 24 h, corresponding to times one, two, and three, respectively). Values are expressed as mean ± SE. Statistically significant differences (*p* < 0.05) between diets within the same time are expressed by letters to represent pairs of diets.

		Time (h)		
		2	5	24
Diets	N20	10.38 ± 1.14	23.29 ± 3.54 a	21.67 ± 2.09
	T20	6.94 ± 0.65	8.01 ± 0.38 a	18.31 ± 3.96
	N40	9.55 ± 1.05	12.67 ± 0.95	18.23 ± 2.03
	T40	9.31 ± 0.94	13.11 ± 1.48	24.11 ± 3.99

**Table 6:** Mean values of food intake (mg diet/g animal) in common cockles (*Cerastoderma edule*) at the different sampling times (2, 5, and 24 h, corresponding to times one, two, and three, respectively). Values are expressed as mean ± SE. Statistically significant differences (*p* < 0.05) between diets within the same time are expressed by letters to represent pairs.

		Time (h)		
		2	5	24
Diets	N20	25.18 ± 4.63 a	25.94 ± 3.36	47.09 ± 6.25 a
	T20	20.02 ± 4.02	14.49 ± 4.48	48.44 ± 9.22 b
	N40	4.86 ± 2.28 a	25.05 ± 3.55	37.15 ± 7.09 c
	T40	14.65 ± 3.33	22.76 ± 4.14	7.24 ± 1.28 a,b,c

## Data Availability

Not applicable.
